# Camellia oil with its rich in fatty acids enhances post-thawed boar sperm quality

**DOI:** 10.1186/s13028-024-00728-y

**Published:** 2024-02-12

**Authors:** Vassakorn Khophloiklang, Panida Chanapiwat, Kampon Kaeoket

**Affiliations:** 1 Semen Laboratory, Department of Clinical Sciences and Public Health, Faculty of Veterinary Science, Mahidol University, Phutthamonthon, Nakhon Pathom, 73170 Thailand; 2 Faculty of Veterinary Science, Rajamangala University of Technology Srivijaya, Thungyai, Nakhon Si Thammarat, 80240 Thailand

**Keywords:** Antioxidant, Boar sperm, Camellia oil, Fatty acids, Freezing

## Abstract

**Background:**

Boar sperm are highly susceptible to specific conditions during cryopreservation, leading to a significant decrease in their fertilizing potential due to damage to their membranes. Camellia oil, known for its fatty acids with antioxidant and biological properties, has not been previously explored for the cryopreservation of boar semen. This study aimed to examine the effects of camellia oil on post-thawed boar sperm quality. Boar semen ejaculates (n = 9) were collected and divided into six equal aliquots based on camellia oil concentrations (0, 0.5, 1, 1.5, 2 and 2.5% v/v) in the freezing extender. Semen samples were processed and cryopreserved using the liquid nitrogen vapor method. Thereafter, frozen semen samples were thawed at 50 °C for 12 s and evaluated for sperm morphology by scanning electron microscope, sperm motility using a computer-assisted sperm analyzer, sperm viability, acrosome integrity, mitochondrial function, MDA level and total antioxidant capacity.

**Results:**

The results demonstrated that the supplementation of 1.5% (v/v) camellia oil showed superior post-thaw sperm qualities such as improved sperm morphology, motility, acrosome integrity and mitochondrial function by 14.3%, 14.3% and 11.7%, respectively, when compared to the control group. Camellia oil at a concentration of 1.5% (v/v) showed the lowest level of MDA (18.3 ± 2.1 µmol/L) compared to the other groups.

**Conclusions:**

In conclusion, adding 1.5% (v/v) camellia oil in the freezing extender reduced the oxidative damage associated with cryopreservation and resulted in a higher post-thawed sperm quality.

## Background

Boar sperm are highly responsive to the conditions encountered during cryopreservation, with several factors coming into play. These factors include susceptibility to oxidative harm, ice formation inside as well as outside the sperm cells during the semen cryopreservation and thawing processes, an overproduction of reactive oxygen species (ROS), and exposure to osmotic stress [1, 2]. It is important to highlight that cryopreservation of boar semen leads to a significant decline in fertilization potential due to the detrimental effects on sperm cell membranes [2]. This is particularly significant because polyunsaturated fatty acids (PUFAs) are abundant in mammalian sperm [3]. Also, the lipid makeup of the sperm's plasma membrane plays a pivotal role in shaping its mobility traits, susceptibility to cold temperatures, viability, and membrane integrity [4]. Notably, boar sperm exhibits a greater abundance of PUFAs within the plasma membrane than other species [5]. The most abundant saturated fatty acids (SFAs) were C16:0 (18%) and C18:0 (16%), and the most abundant fatty acids were docosapentaenoic Acid (DPA) (15%) and docosahexaenoic acid (DHA) (16%) [6]. Fatty acids (FAs) are important in male sperm biology due to their close association with membrane fluidity, acrosome reaction, sperm motility, and viability [7]. Within sperm membranes, FAs play a pivotal role in shaping both the structure and function of sperm, facilitating crucial membrane fusion events during fertilization [4]. Among these FAs, PUFAs stand out for their ability to permeate the sperm cell membrane, bolster the flexibility of the sperm plasma membrane, uphold its structural and functional integrity, bolster the resistance of the acrosome membrane to osmotic stress, and provide safeguarding against physiological or thermal fluctuations encountered during cryopreservation [3, 8, 9]. Nevertheless, diminishing the presence of PUFAs within the sperm plasma membrane can trigger oxidative stress, suggesting a connection between lipid peroxidation and sperm motility, viability, and morphology [10]. To protect sperm cells from the oxidative stress induced by free radicals, a robust array of antioxidants and fatty acids has been used in semen cryopreservation. It has been reported earlier that those fatty acids from various sources, such as DHA from fish oil [11–13], DHA from different egg yolks, and olive oil, [14] influence fatty acids on the sperm plasma membrane and consequently improve post-thawed boar semen quality [15]. Besides supplementation of fatty acid into freezing extender, palm kernel meal protein hydrolysate with its bioactive peptides [16, 17], curcumin [18], and resveratrol [19] have also been applied to improve frozen boar semen quality, such as total motility, progressive motility and viability as well as minimize lipid peroxidation.

Camellia oil, also known as tea seed oil, is a versatile vegetable oil that is extracted from the seeds of the *Camellia oleifera* plant or the *Camellia sinensis* plant, which is the same plant used to make tea [20]. It has been traditionally used in various Asian cuisines, and there are trends known to all regions as applications in skincare and haircare products due to its potential benefits [21]. Camellia seed oil, which is also called oriental olive oil, is recommended as a health-care plant oil by the FAO because of its high content of unsaturated fatty acids, polyphenols, vitamin E, and carotene [21]. Camellia oil is rich in unsaturated fatty acids such as oleic acid, linoleic acid; sesamin, saturated acids, and polyphenols [22–24]. Many studies reported that camellia oil with its rich in FAs and other advantage compounds, has a variety of bioactivities including antioxidant, anti-inflammatory, antimicrobial, gastroprotective, and hepatoprotective bioactivity [25]. During the past decades, numerous studies demonstrated that oleic [26, 27], linoleic [28, 29], vitamin E [30], and polyphenol [31], which are also constituents in camellia oil, could improve sperm qualities and reduce lipid peroxidation during cryopreservation of boar semen and other species. However, no studies have been published on the effects of camellia oil during the cryopreservation of boar sperm. This study, therefore, aims to elucidate the cryoprotection effect of camellia oil by evaluating the post-thawed boar semen qualities.

## Methods

### Animals

A total of nine semen ejaculates (*n* = *9*) were obtained from six boars, comprising both Landrace and Large White breeds, with ages ranging from 1.5 to 3 years old, from a commercial farm in Ratchaburi province, Thailand were used in this study. These boars were individually housed in pens equipped with an evaporative cooling system. They had access to fresh, clean water at all times through automated watering systems, and their daily feed intake was adjusted to fulfil the semen production demands, which amounted to 3 kg per day. These boars were regularly utilized for semen collection for the purpose of artificial insemination.

### Chemicals and extenders

In this study, the commercial oil named camellia oil from the Thailand Royal Project (Camellia oleifera seed oil, FDA No. 57-2-03254-2-0001, PatPat, Bangkok, Thailand) was used. There were three extenders used for boar semen cryopreservation as follows: extender I was the commercial semen extender the Beltsville Thawing Solution (BTS, Minitube, Tiefenbach, Germany); extender II was composed of 20% egg yolk and 11% lactose solution supplemented with different concentrations of camellia oil solution (0, 0.5, 1, 1.5, 2, and 2.5% v/v) in distilled water. Each aliquot was diluted with or without (control) camellia oil substance with an equal extender volume; and extender III was composed of 89.5% extender II, with 9% (v/v) glycerol and 1.5% (v/v) Equex-STM^®^ (Nova Chemical Sales Inc., Scituate, MA, USA).

### Fatty acid determination

The fatty acid profiles of camellia oil were determined by followed the Association of Official Analytical Chemists 2005 (AOAC) [15]. The content of the total fat for 32 fatty acids was determined by calculating the area under the peak and presented as each fatty acid per 100 g of total fatty acid.

### Semen collection and preparation

Boar semen samples were obtained using the glove-hand technique. Subsequently, the semen was passed through gauze to retain only the sperm-rich fraction, which was then subjected to a comprehensive evaluation based on various parameters, including semen volume, pH, sperm motility, concentration, sperm viability, and morphologically intact spermatozoa (sperm morphology was stained with William’s staining method and evaluated using a light microscope). For cryopreservation process, only sperm-rich fraction with a motility of at least 70% and a morphology of at least 80% were selected [32].

### Semen freezing and thawing process

Each sperm sample underwent cryopreservation using the conventional nitrogen freezing technique. In brief, post-collection, the semen was diluted with the BTS extender in a 1:1 volume ratio. The diluted semen was then transferred into 50 mL centrifuge tubes and subjected to a stabilization period at 15 °C for 120 min. After this equilibration period, the samples were centrifuged at 15 °C, with a force of 800 g applied for 10 min. (LMC-4200R, Biosan, Latvia) After centrifugation, the supernatant was removed, and the sperm pellet was resuspended at a concentration of 1.5 × 10^9^ sperm/mL in extender II at an approximate ratio of 1–2:1. At this stage, the sperm sample was partitioned into six groups, distinguished by varying concentrations of camellia oil (0, 0.5, 1, 1.5, 2, and 2.5% v/v) and subsequently, cooled to 5 °C for 90 min. Each group of samples was then blended with extender III to attain a concentration of 1.0 × 10^9^ sperm/mL before being loaded into 0.5 mL straws (IMV Technologies, L'Aigle, Basse-Normandie, France). The semen straws were frozen using a traditional nitrogen by contacting nitrogen vapor at 4 cm above the liquid nitrogen level for 20 min (−  20 °C /min) in a polystyrene box and plunged into the liquid nitrogen tank (− 196 °C) for storage prior to analysis. The frozen semen had been kept for 12 h before evaluations. Before sperm evaluation, the frozen semen samples were thawed at 50 °C for 12 s and extended (1:6) with a pre-warmed BTS extender at 37 °C for 15 min [33].

### Assessment of sperm motility

Sperm motility assessments were performed using Computer-assisted Sperm Motility Analysis (CASA) with the AndroVision^®^ system (Minitube, Tiefenbach, Germany). In brief, 3 µL of the semen sample was meticulously transferred into a disposable counting chamber (Leja^®^ 20 µM, IMV Technologies, L’Aigle, Basse-Normandie, France) and maintained at a constant temperature of 37 °C during the entire analysis. Each analysis involved the enumeration of a minimum of 600 sperm cells, derived from examining five different fields within each sample. The results were expressed as percentages, reflecting total sperm motility, progressive motility, and various motility parameters, which encompassed curvilinear velocity (VCL, µm/s), average pathway velocity (VAP, mm/s), straight-line velocity (VSL, mm/s), amplitude of lateral head displacement (ALH, mm), straightness (STR, %), and linearity (LIN, %). Motile spermatozoa were defined with VCL ≥ 24 µm/s and ALH > 1 µm. Progressive motility (PMOT) has been interpreted as the presence of a VCL ≥ 48 µm/s and a VSL < 10 µm/s. The total motility (MOT) is the summation of sperm motility subpopulations that were determined by VCL thresholds, including local motility (VCL ≥ 24 and < 48 µm/s), slow motility (VCL ≥ 48 and < 80 µm/s), and fast motility (VCL ≥ 80 µm/s) [16].

### Assessment of sperm viability

Sperm viability was evaluated utilizing a staining procedure employing SYBR-14 (L7011(A); Live/Dead^™^ Sperm viability kit, Invitrogen, Waltham, MA, USA) and Ethidiumhomodimer-1 (EthD-1, E1169, Invitrogen, Waltham, MA, USA). In brief, 50 µL of semen was mixed with 2.7 µL of 0.54 µM SYBR-14 working solution in DMSO to arrive at a final SYBR 14 concentration of 0.27 µM and 10 µL of 4.68 µM EthD-1 was added to the sample to yield a final EthD-1 concentration of 2.34 µM. The resulting mixture was incubated at 37 °C for a duration of 15 min. a fluorescence microscope magnified at 400 × , 200 sperm were assessed. The SYBR-14/EthD-1 stained sperm were classified into viable and non-viable sperm. Nuclei from viable sperm with intact plasma membranes exhibited a green fluorescence, whereas those from deceased sperm or sperm with damaged plasma membranes displayed a red fluorescence. The percentage of viable and non-viable sperm were calculated [33].

### Assessment of acrosome integrity

**Fig. 1:**
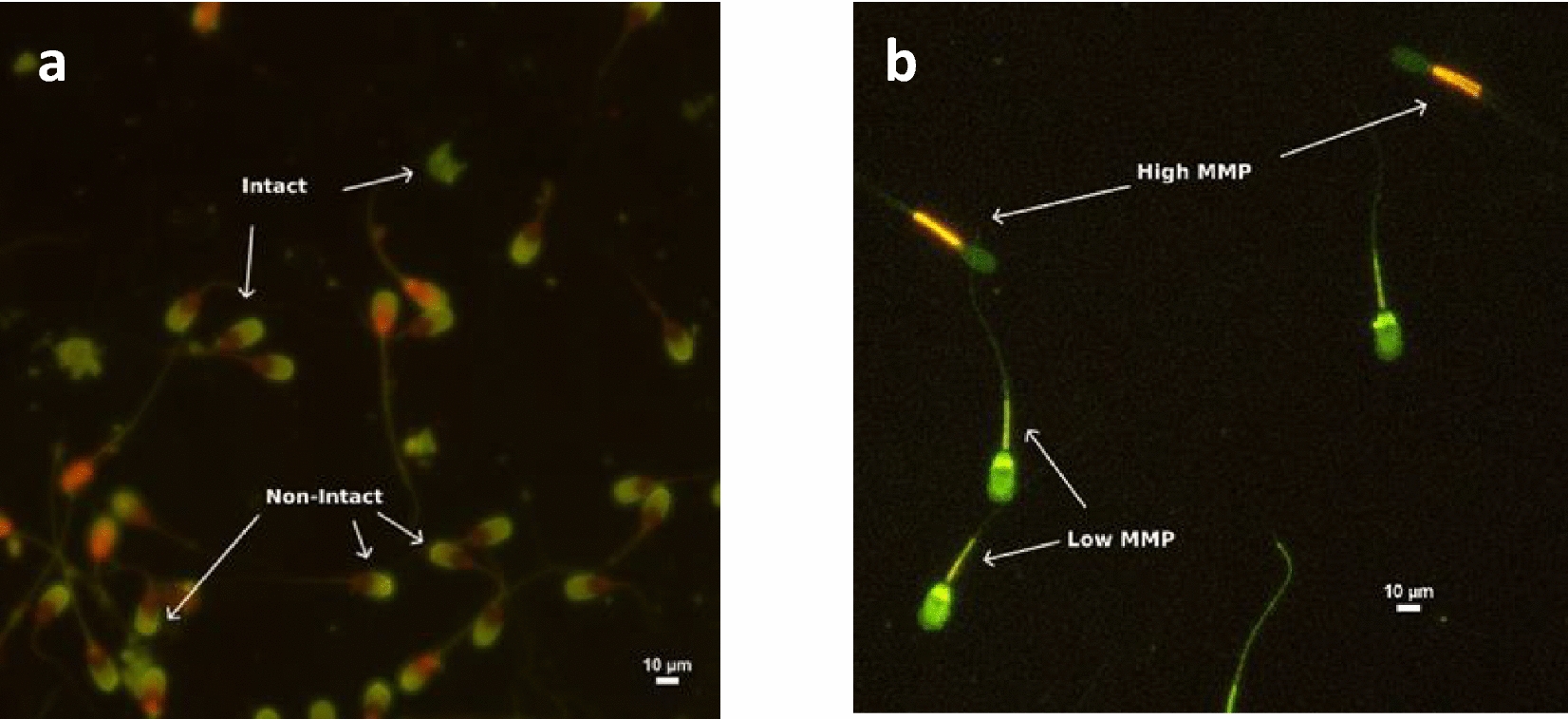
Assessment of frozen-thawed boar spermatozoa using specific fluorescent dyes (400 × magnification). **a** Intact and non-intact acrosomes stained with FITC-PNA/EthD-1 staining (**b**) Mitochondrial membrane potential (MMP) stained with the dyes JC-1 and PI (counted only viable sperm); high MMP with orange fluorescence in midpiece; and low MMP with green fluorescence in midpiece

Acrosome integrity was assessed through fluorescein isothiocyanate-labelled peanut (*Arachis hypogaea*) agglutinin (FITC-PNA; L7381, Sigma-Aldrich Co., Darmstadt, Germany). staining. Specifically, 10 µL of diluted semen was combined with 10 µL of 4.68 µM EthD-1. The sample was processed to obtain a final concentration of EthD-1 of 2.34 µM and incubated at 37 °C for 15 min. Following this, a 5 µL sample was smeared onto a glass slide and allowed to air dry. Subsequently, the sample was fixed with 95% ethanol for 30 s and air-dried. Forty µL of FITC-PNA (100 µg/mL in PBS) was evenly distributed over the slides, which were then placed in a moist chamber at 4 °C for 30 min. The slides were subsequently rinsed with cold PBS and air-dried once more. Under a 1000 × magnification fluorescence microscope, a total of 200 sperm cells were examined. Sperm with intact acrosomes exhibited a green colour with a smooth contour on the acrosomal region, as depicted in Fig. 1. Conversely, sperm with damaged acrosomes displayed a green colour with a rough contour. The results were quantified as the percentage of sperm with intact acrosomes [33].

### Assessment of mitochondrial membrane potential

The mitochondrial membrane potential was evaluated using a staining protocol employing the fluorochrome 5,5′,6,6′-tetrachloro-L,L′-tetraethylbenzimidazolylcarbo cyanine iodide (JC-1, T3168, Invitrogen, Waltham, MA, USA). A total of 50 µL of diluted semen was combined with 3 µL of a 2.4 mM propidium iodide (PI) (L7011(B); Live/Dead™ Sperm viability kit, Invitrogen, Waltham, MA, USA) working solution in to arrive at a final PI concentration of 129 µM and 3 µL of a 1.53 mM JC-1 solution in DMSO was added to the sample to yield a final JC-1concentration of 82 µM. This mixture was then incubated for 10 min at 37 °C in an opaque container. Under a 400 × magnification fluorescence microscope, 200 viable sperm (PI-neg) were assessed. Midpiece staining revealed that sperm exhibiting a heightened mitochondrial membrane potential emitted a yellow-orange fluorescence, while sperm with a diminished membrane potential emitted a green fluorescence (Fig. 1). The percentage of sperm with a high mitochondrial membrane potential was calculated [34].

### Assessment of lipid peroxidation

The levels of malondialdehyde (MDA) in the semen samples were quantified using the concentration of MDA using a colorimetric lipid peroxidation assay kit (OxiSelect™ TBARS Assay Kit, Cell Biolabs, Inc, San Diego, USA) following the manufacturer’s instructions. In brief, 250 µL of a post-thawed semen sample was lysed by freezing at − 20 °C for 12 h and then centrifuged at a speed of 20000×*g* for 10 min. The supernatant was collected and analysed. The MDA in the supernatant sample (100 µL) reacted with 100 µL of lysis solution and 250 µL of thiobarbituric acid (TBA) solution to generate an MDA-TBA adduct that was quantified. The MDA-TBA product was measured immediately in a microplate reader (SPECTROstar Nano, BMG LABTECH, Ortenberg, Germany) at 532 nm. For the colorimetric assay, a 2 mM MDA standard was prepared and serially diluted for the standard curve. The levels of MDA were calculated from the MDA standard curve and expressed as µmol/L [16].

### Assessment of total antioxidant capacity

The total antioxidant capacity (TAC) was monitored by using a colorimetric total antioxidant capacity assay kit (ab65329, Abcam^®^, Cambridge, UK) following the manufacturer’s instructions. Briefly, 250 µL of post-thawed sperm in each treatment was centrifuged at 20000 *g* for 10 min, supernatant was collected and diluted (1:100) in double distilled water. The 100 µL of diluted samples and standards were added to the 96 well plate. All standards and samples were mixed with 100 µL of Cu^2+^ solution and, incubated at room temperature for 90 min. After incubation, the samples were measured immediately in a microplate reader (SPECTROstar Nano, BMG LABTECH, Ortenberg, Germany) at 570 nm. For the colorimetric assay, a 1 mM Trolox standard was prepared and serially diluted for the standard curve. The TAC was calculated from the TAC standard curve and expressed as µmol/L.

### Evaluation of sperm morphology by scanning electron microscopy (SEM)

Sperm samples were evaluated for morphology under a scanning electron microscope using the typical usual approach as follows: the semen samples were preserved in PBS for 24 h with 2.5% glutaraldehyde (Electron Microscopy Sciences, UK). Following fixation, the washing process with PBS was repeated three times for 15 min each. After staining with 0.1% osmium tetroxide (Sigma-Aldrich, Germany) for 1 h, the samples were rinsed three times for 15 min with PBS. The samples were dehydrated with a graded series of ethanol concentrations of 70%, 80%, 90%, 95%, and 100% ethanol during the dehydration process. The sperm samples were treated and subsequently coated with 50 nm platinum particles on a SEM stub [35]. Finally, the morphology of the sperm was examined using a scanning electron microscope (JEOL, JSM-IT500LA, Japan).

### Statistical analysis

Statistical analysis was performed using IBM SPSS Statistics for Windows, version 26.0 (SPSS Inc., Chicago, IL, USA). The normal distribution test of the data was examined using the Shapiro–Wilk test; the sperm parameters (i.e., progressive motility and VAP) were not normally distributed, and were then transformed using the Log 10 transformation. The parameters, including total motility, progressive motility, sperm motility patterns, sperm viability, acrosome integrity, mitochondrial membrane potential, MDA levels, and total antioxidant capacity, were presented as mean ± SEM. Owing to the main aim of this experiment was to assess the cryoprotective effect of varied concentrations of camellia oil on post-thawed boar semen qualities, and not for the variation among individual boar or different breeds or boar within the same breed. Therefore, the homogeneity of variances was tested to investigate the effect of treatment by Levene’s test, and means were compared using a one-way ANOVA. The comparison of sperm parameters among treatment groups was performed by Duncan’s multiple range test. Statistically significant difference was defined as P < 0.05.

## Results

### Effects of camellia oil on sperm motility

**Fig. 2:**
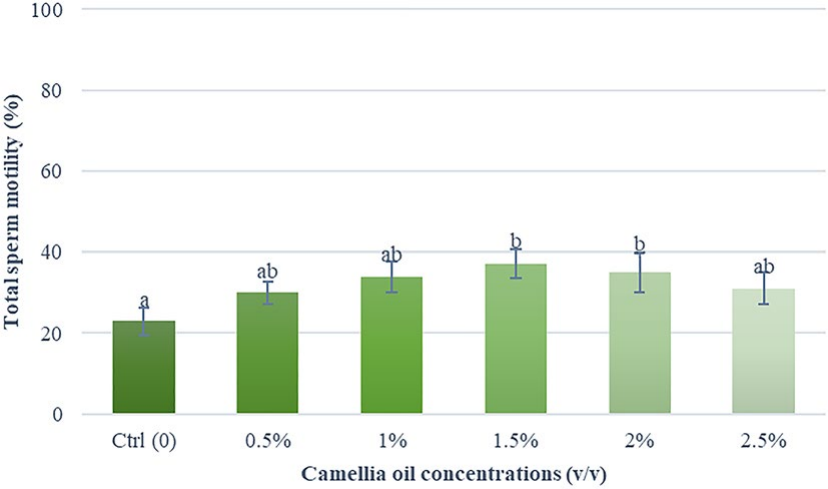
Effect of camellia oil on total sperm motility in post-thawed boar semen. Bars represent means ± SEM. Different letters indicate a statistically significant difference at P < 0.05

**Fig. 3:**
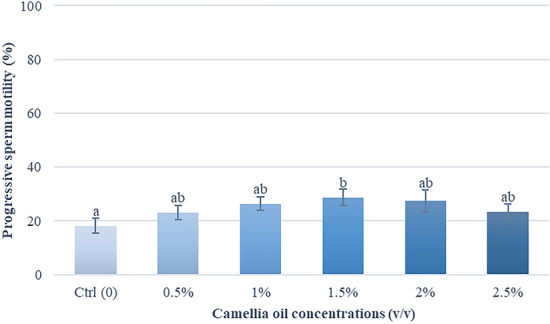
Effect of camellia oil on progressive sperm motility in post-thawed boar semen. Bars represent means ± SEM. Different letters indicate a statistically significant difference at P < 0.05

Table 1 presented descriptive statistics related to the quality of fresh boar semen. All the measurements affirm that the quality of these fresh semen samples falls within acceptable standards. Figures 2 and 3 illustrated the impact of camellia oil on sperm motility. The findings indicate that a 1.5% (*v/v*) supplementation of camellia oil exhibited superior post-thaw sperm quality in comparison to other concentrations. Semen samples supplemented with 1.5% (*v/v*) improved total motility and progressive motility by 14.3% and 10.5% when compared with control (37.2 ± 3.6% vs 22.9 ± 3.2% and 28.7 ± 3.0% vs 18.2 ± 2.8%, respectively). A significantly higher percentage of sperm characteristics of movement (i.e. VCL, VSL, VAP, and ALH) was found in 1.5 and 2% (*v/v*) supplementation groups when compared with the control group (Table 2).

**Table 1:** Sperm quality of fresh boar semen (*n* = *9*)

Parameters*	Mean ± SEM	Range
Concentration (× 10^6^ sperm/mL)	203.7 ± 24.2	100–345
Total motility (%)	93.4 ± 1.1	91–98
Progressive motility (%)	86.7 ± 2.1	81–96
Sperm viability (%)	89.5 ± 0.7	86–92
Acrosome integrity (%)	86.3 ± 2.1	79–94

* Results are expressed as mean ± SEM (*n* = 9)

**Table 2:** Sperm motility parameters after thawing at different camellia oil concentrations

Parameters^*^			Concentrations (% *v/v*)				
	Ctrl (0)	0.5	1	1.5		2	2.5
VCL	28.8 ± 3.3^b^	38.4 ± 3.2^ab^	40.2 ± 3.8^ab^	43.6 ± 4.0^a^		43.1 ± 5.5^a^	33.3 ± 3.7^ab^
VSL	10.5 ± 1.5^b^	13.1 ± 1.4^ab^	14.9 ± 1.6^a^	16.3 ± 1.8^a^		16.5 ± 2.6^a^	12.6 ± 1.8^ab^
VAP	13.8 ± 1.7^b^	18.2 ± 1.9^ab^	19.5 ± 1.8^ab^	21.4 ± 2.2^a^		21.3 ± 3.1^a^	16.4 ± 2.1^ab^
ALH	0.3 ± 0.03^b^	0.47 ± 0.02^ab^	0.50 ± 0.04^a^	0.50 ± 0.02^a^		0.50 ± 0.04^a^	0.41 ± 0.03^ab^
STR	71.6 ± 3.5^a^	69.1 ± 1.6^a^	73.0 ± 2.2^a^	74.4 ± 1.4^a^		73.3 ± 2.2^a^	73.3 ± 2.3^a^
LIN	36.0 ± 2.6^a^	33.8 ± 1.4^a^	38.1 ± 2.0^a^	37.0 ± 1.3^a^		37.0 ± 1.6^a^	37.2 ± 1.8^a^

* Results are expressed as mean ± SEM (*n* = 9).^a, b, c^ Means with different superscripts in the row are significantly different between groups (P < 0.05) P-MOT: progressive motility (%), VCL (µm/s): curvilinear velocity, VSL (µm/s): velocity straight line, VAP (µm/s): average pathway velocity, ALH (µm): amplitude of lateral head displacement, STR (%): straightness, LIN (%): Linearity

### Effects of camellia oil on sperm viability

**Fig. 4:**
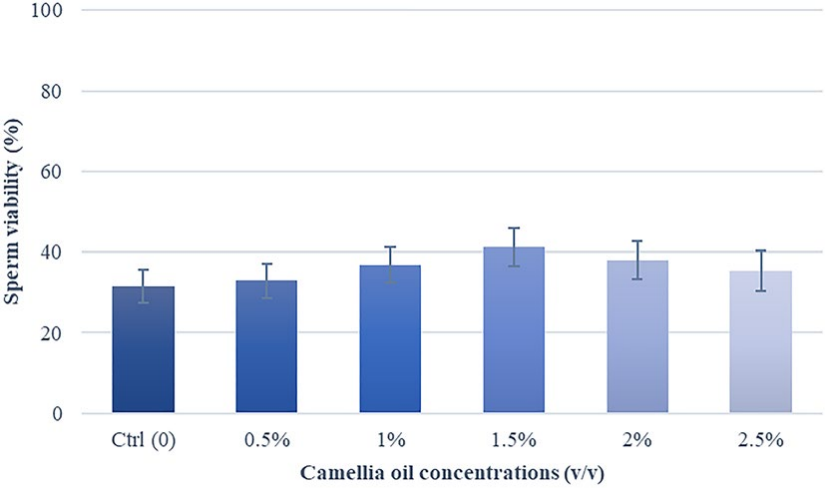
Effect of camellia oil on sperm viability after thawing. Bars represent means ± SEM. There are no statistically significant differences (P > 0.05)

There was no statistically significant difference in sperm viability (P = 0.19). However, a tendency toward a higher percentage of viability in treatment groups than the control group was found (Fig. 4). Supplemented with 1.5% (*v/v*) camellia oil showed the highest viable sperm (41.3 ± 4.7%) and was higher than control by 9.8%.

### Effects of camellia oil on sperm acrosome integrity

**Fig. 5:**
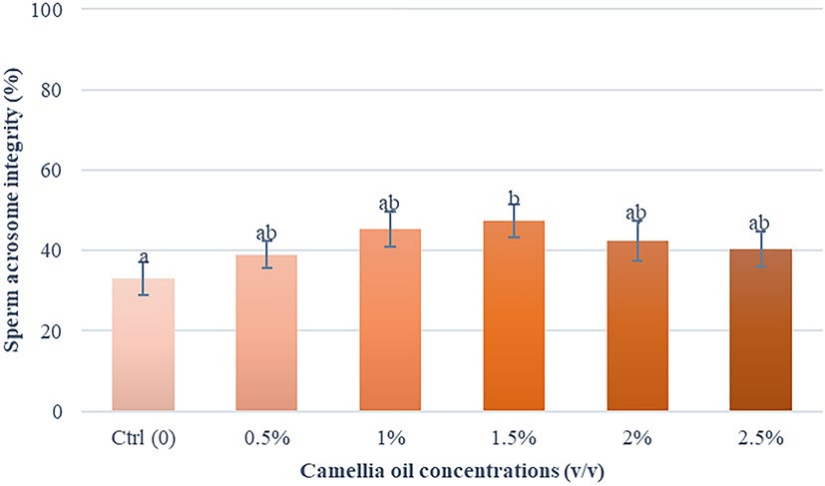
Effect of camellia oil on acrosome integrity after thawing. Bars represent means ± SEM. Different letters indicate a statistically significant difference at P < 0.05

A variation in acrosome integrity was observed among the control and treatment groups (Fig. 5). However, the highest percentage of acrosome integrity was found at a concentration of 1.5% (*v/v*) (47.4 ± 4.2%), which was significantly higher than the control group by 14.3%.

### Effects of camellia oil on mitochondrial membrane potential

**Fig. 6:**
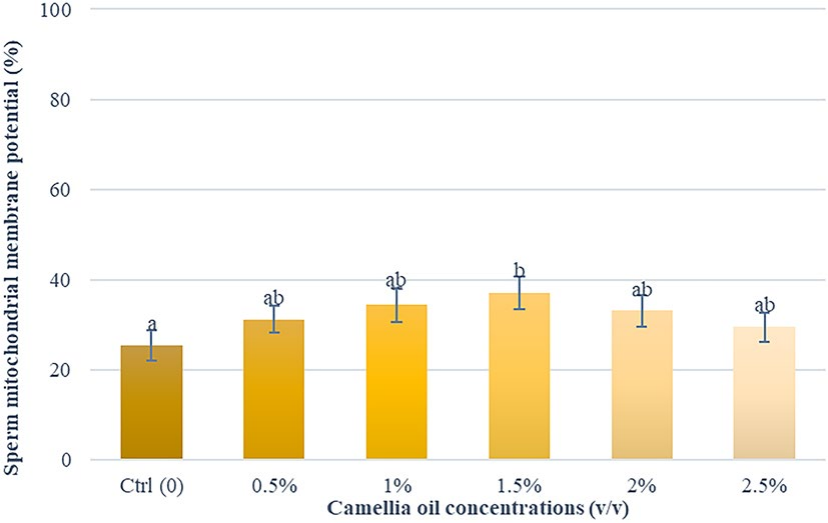
Effect of camellia oil on mitochondrial membrane potential after thawing. Bars represent means ± SEM. Different letters indicate a statistically significant difference at P < 0.05

The results of mitochondrial membrane potential are presented in Fig. 6. A significantly higher percentage of mitochondrial function in 1.5% (*v/v*) supplemented group (37.1 ± 3.5%) was found when compared with the control group.

### Effects of camellia oil on lipid peroxidation

**Fig. 7:**
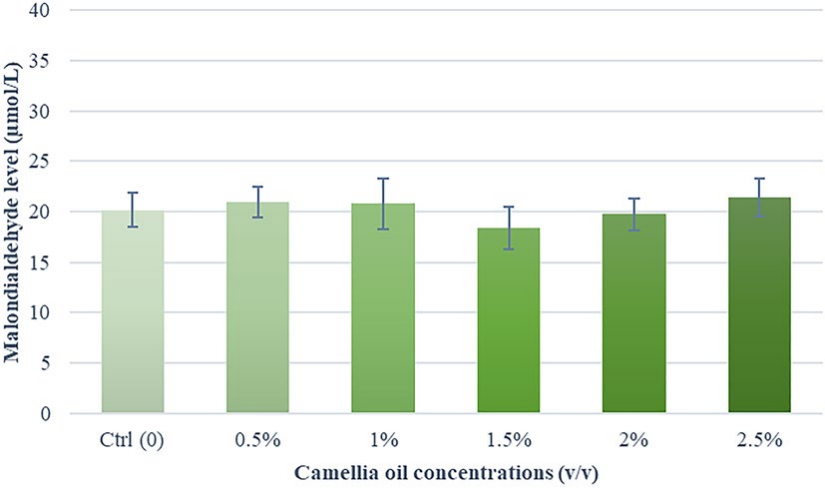
Effect of camellia oil on the lipid peroxidation (MDA levels) after thawing. Bars represent means ± SEM. There are no statistically significant differences (P > 0.05)

Figure 7 illustrates the effect of camellia oil on lipid peroxidation during cryo- preservation. It indicated a trend toward lower MDA levels in the 1.5% and 2.0% (*v/v*) supplemented group compared to the control group (P = 0.3).

### Effects of camellia oil on total antioxidant capacity

**Fig. 8:**
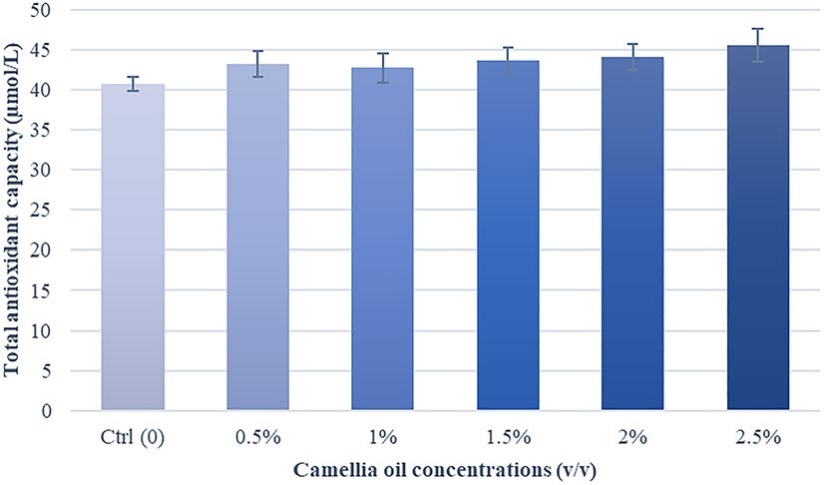
Effect of camellia oil on the total antioxidant capacity (TAC) after thawing. Bars represent means ± SEM. There are no statistically significant differences (P > 0.05)

As depicted in Fig. 8, there was no significant difference between the treatments and control groups. However, there was a trend for increased TAC in the treatment groups when compared to the control group (P = 0.08).

### Effects of camellia oil on sperm morphology

**Fig. 9:**
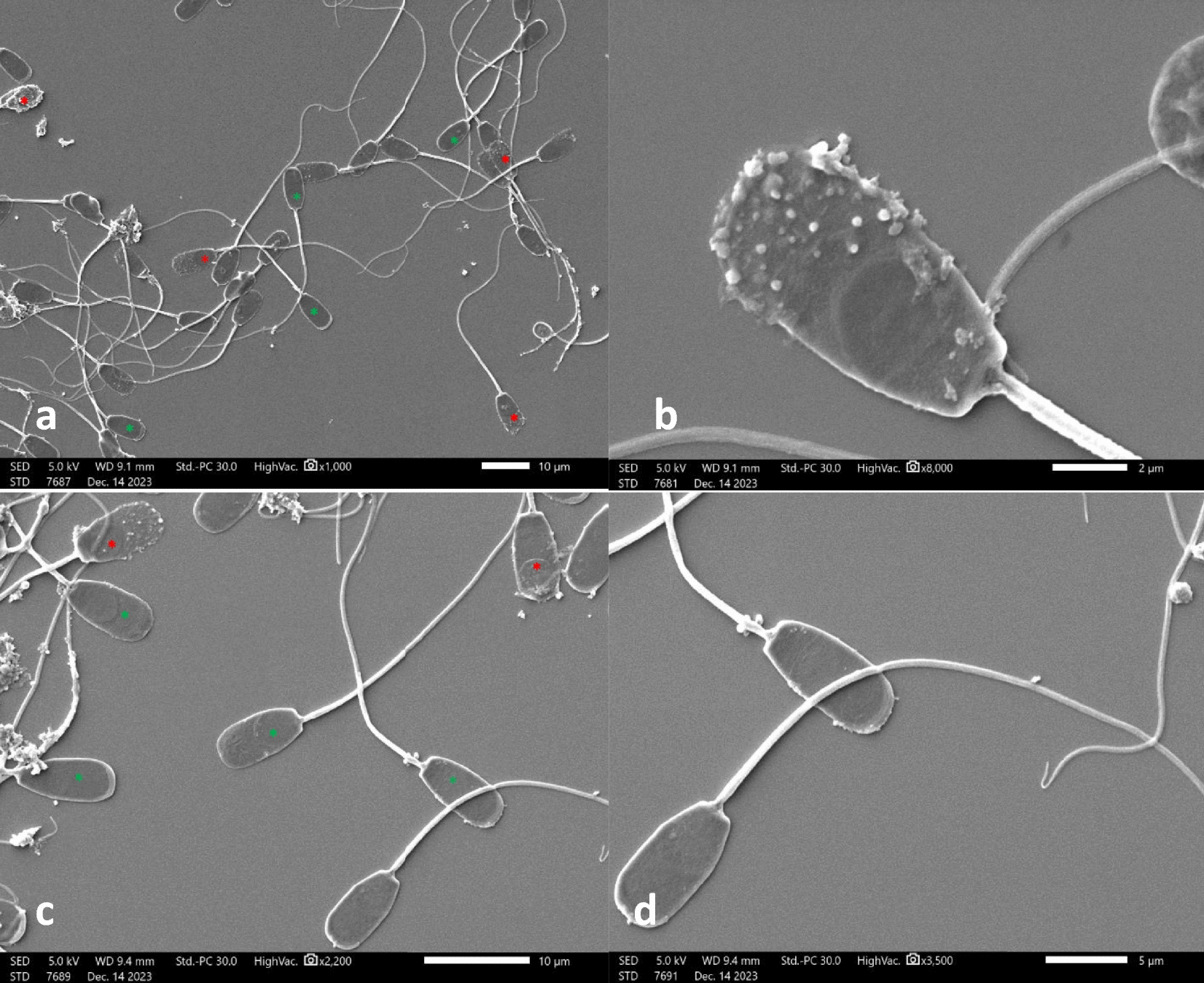
Scanning electron micrographs of post-thawed boar sperm. The post-thawed boar sperm without supplementation (control group): (**a**, 1,000X) showed a high number of non-intact sperm morphology; (**b**, 8,000X) showed acrosome damage on the acrosome region. The post-thawed boar semen with 1.5% of camellia oil supplement: (**c**, 2,200X) showed a high number of intact sperm morphology; (**d**, 3,500X) showed intact sperm morphology. The sperms (**a**, **c**) with intact morphology were marked with a green star; sperms with non-intact morphology were marked with a red star

The sperm morphology evaluation using scanning electron microscopy (SEM) are presented in Fig. 9. The sperm morphology in the control group (Fig. 9 a, b) showed a higher number of non-intact morphology, including plasma membrane and acrosomal damage, while those supplemented with 1.5% camellia oil (Fig. 9 c, d) showed a high number of intact sperm morphology with a lesser degree of abnormal morphology than those in the control group.

### Fatty acid determination

From 32 fatty acids determination, the omega 6, 9, palmitic acid and stearic acid are more pronounced in the composition of fatty acids in camellia oil and a high ratio of omega 6:3 was found (Table 3).

**Table 3:** Fatty acids composition of camellia oil

Type of fatty acid	g/100 g of total fatty acid
C16:0 (Palmitic Acid) (Saturated fatty acids)	11.12
C18:0 (Stearic Acid) (Saturated fatty acids)	2.43
C18:1n9c (Oleic Acid: Omega 9) (Monounsaturated fatty acids)	78.05
C18:2n6c (Linoleic Acid: Omega 6) (Polyunsaturated fatty acids)	8.08
C18:3n3 (Linolenic Acid: Omega 3) (Polyunsaturated fatty acids)	0.32
Ratio	
Omega 6: Omega 3 fatty acids	25.25
Saturated fatty acids: Unsaturated fatty acids	0.16
Polyunsaturated fatty acids: Saturated fatty acids	0.61

## Discussion

In the course of cryopreserving mammalian sperm, the freezing and thawing procedures, referred to as cryoinjuries, induce oxidative stress and an excessive production of ROS. This, in turn, disrupts the balance of the antioxidant system, where antioxidant defences struggle to counteract the overabundance of ROS. Consequently, this imbalance has a cascading impact on semen qualities, including sperm motility, mitochondrial activity, membrane permeability, sperm functions, and ultimately, sperm survival rates [36, 37].

The results of the first report of camellia oil with optimal concentration in freezing boar semen clearly showed that adding camellia oil to the freezing extender significantly improved post-thawed boar semen parameters, including sperm morphology by SEM, total motility (14.3%), progressive motility (10.5%), acrosome integrity (14.3%), mitochondrial membrane potential (11.7%) and kinetic motility patterns such as VCL, VSL, VAP and ALH. In the present results, an increase in sperm viability, total antioxidant capacity and inferior level of MDA, indicating a lesser level of lipid peroxidation, found in the treatment groups than in the control, particularly when supplemented with 1.5% (*v/v*) of camellia oil concentration. This might be explained by the observation that spermatozoa absorbed and employed fatty acids from the freezing extender to counteract ROS [37]. As a result, this process led to a reduction in lipid peroxidation within their plasma membrane and internal organelles [38]. The substantial variability observed in the parameters within the current study could potentially be attributed to individual variations among boars, encompassing distinctions in sperm biochemistry, seminal plasma composition, physiological factors, polymorphisms in the testis and epididymis, as well as the presence of fatty acids, for example, palmitic acid (16:0), stearic acid (18:0), oleic acid (18:1, n-9), docosapentaenoic acid (22:5, n-6) and docosahexaenoic acid (22:6, n-3) in the boar sperm plasma membrane which has been earlier reported [6]. This relationship between PUFAs, FAs and cryopreservation susceptibility has been previously reviewed [36]. Considering all the findings collectively, it is concluded that the inclusion of 1.5% (*v/v*) camellia oil represents the optimal concentration for cryopreserving boar semen. This finding suggests that fatty acids, due to their antioxidant properties, exert a protective influence on spermatozoa during the freezing process, thereby mitigating damage to their membrane, acrosome, and mitochondria. In agreement with our study, it has been reported that oleic acid supplement in rooster chilled extender improved semen quality, decreased MDA levels and increased TAC [27]. In addition, it has been documented that oleic acid and palmitic acid improved sperm motility, viability, acrosomes, mitochondrial membrane potential and ATP production in chilled boar sperm [26]. According to previous studies in boar [33] and another study in buffalo [39], fatty acid antioxidants such as omegas 3, 6, 9 and DHA improved post-thawed semen quality in terms of plasma membrane integrity and acrosome integrity. It has also been reported by many studies that supplementation of omega 3 and/or 6 in feed improve not only semen qualities in boar but also reproductive performance in sows [38, 40–44]. In this study, camellia oil with its high constituent of omega 6, 9, palmitic acid and stearic acid also revealed its antioxidant capacity by protecting sperm from cryodamage associated with sperm freezing and thawing. However, too high a concentration of camellia oil showed a slightly cytotoxic effect on sperm qualities, which have also been reported in other antioxidants [45, 46]. The reason might be that an excess of antioxidants may disturb the balance between free radicals and antioxidants in mammalian cells [47].

The camellia oil in this study contained palmitic acid, stearic acid, oleic acid, linoleic acid, linolenic acid, and saturated fatty acids as 11.12, 2.43, 78.05, 8.08, 0.32, and 13.55 g/100 g of total fatty acids, respectively. According to previous studies [22, 24], they showed that in camellia oil, more than 76% of the fatty acids are oleic acid and 50% higher than those compared with olive oil. In addition, it has also shown that camellia oil has antioxidant, anti-inflammatory, antimicrobial, gastroprotective, and hepatoprotective bioactivity [25]. Many studies have revealed excellent antioxidant activities of camellia oil, such as researchers in China, who demonstrated that the methanol extract of tea seed oil exhibited the highest yield and the strongest antioxidant activity as determined by DPPH scavenging activity and trolox equivalent antioxidant capacity (TEAC) [48]. Moreover, there is another study presented that the IC_50_ values of tea seed oil extracted by the DPPH free radical scavenging assay ranged from 17.2 ± 0.1% to 93.4 ± 1.8% when the concentration of the extracted oil varied from 10 to 160 mg/mL [49]. However, it is important to recognize that an abundance of antioxidants may interfere with redox signalling pathways, thereby disrupting normal cell function. Over-suppression of antioxidant enzyme genes or elevated antioxidant levels can upset the delicate balance of cellular homeostasis, possibly resulting in adverse effects on cellular health [50, 51]. This observation is clarified by the current findings, which demonstrate that incorporating the optimal 1.5% (*v/v*) concentration of camellia oil into the freezing extender improved the quality of post-thawed semen. Conversely, if a concentration exceeding 2% (*v/v*) is added, it leads to a decrease in post-thawed semen quality. However, camellia oil is a natural product and might be difficult to standardize for use as a semen extender additive. Therefore, each batch of camellia oil has to be analysed for its fatty acids composition prior using in each particular experiment. The conducting additional research involving fertility tests with frozen-thawed sperm supplemented with camellia oil for artificial insemination on pig farms would not only generate greater interest, but also provide substantial credibility to the swine industry, ultimately enhancing fertility rates.

## Conclusions

Considering the cumulative results, it can be concluded that camellia oil, owing to its fatty acids content, effectively diminishes ROS production during cryopreservation. This reduction in ROS contributes to the inhibition of lipid peroxidation. It enhances various aspects of sperm quality, including sperm morphology, motility, viability, intact acrosomes, mitochondrial membrane potential, and total antioxidant capacity in frozen-thawed boar sperm. Optimal supplementation of camellia oil at a concentration of 1.5% (*v/v*) within the freezing extender significantly enhances post-thawed boar semen quality. Conversely, it is possible that supplementing with more than 2% (*v/v*) have a negative impact on post-thawed semen quality.

## Data Availability

The datasets used and/or analysed during the current study are available from the corresponding author on reasonable request.
